# Management of Bleeding in Post-liver Disease, Surgery and Biopsy in Patients With High Uncorrected International Normalized Ratio With Prothrombin Complex Concentrate: An Iranian Experience

**DOI:** 10.5812/ircmj.12260

**Published:** 2013-12-05

**Authors:** Alireza Parand, Naser Honar, Khashayar Aflaki, Mohammad Hadi Imanieh, Mahmood Haghighat, Nader Cohan, Sezaneh Haghpanah, Marco Marietta, Zahra Serati, Saeedeh Haghbin, Mehran Karimi

**Affiliations:** 1 Iranian Hospital, Dubai, UAE; 2 Hematology Research Center, Shiraz University of Medical Sciences, Shiraz, IR Iran; 3 Pediatrics Gastroenterology Department, Shiraz University of Medical Sciences, Shiraz, IR Iran; 4 Pediatric Intensive Care Unit, Shiraz University of Medical Sciences, Shiraz, IR Iran; 5 Department of Hematology, University Hospital of Modena, Modena, Italy

**Keywords:** Prothrombin Complex Concentrates, Liver Diseases, General Surgery, International Normalized Ratio

## Abstract

**Background:**

To evaluate the efficacy of prothrombin complex concentrate (PCC) in the management of bleeding in patients with liver disease and patients undergoing surgery or biopsy who had a high uncorrected international normalized ratio (INR).

**Objectives:**

In this study, we examined an Iranian sample and investigated the efficacy of PCC to manage bleeding in patients with liver disease and also patients with high uncorrected INR who were scheduled for surgery or biopsy.

**Materials and Methods:**

A total of 25 patients including 16 patients with post-liver disease bleeding (group 1) and 9 patients with high uncorrected INR who were scheduled for surgery or biopsy (group 2) were enrolled. All patients were treated with 25 IU/kg PCC, and efficacy was defined as any reduction in or cessation of bleeding episodes and correction of INR before surgery or biopsy. The patients were also evaluated for any adverse effects.

**Results:**

INR decreased significantly in both groups of patients, with no bleeding episodes during or after the study in group 1 and during or after surgery/biopsy in group 2. All patients tolerated the therapy well without any significant adverse effects.

**Conclusions:**

The efficacy of PCC therapy was satisfactory in this study. PCC therapy in patients with liver disease and patients undergoing surgery or biopsy seems to be effective and safe, and may be a good treatment strategy for these patients, if fresh frozen plasma or vitamin K are not effective.

## 1. Background

Most coagulation factors are synthesized by the liver, and as a result bleeding in patients with liver disease, as well as in patients who undergo surgery, is an important medical problem. Restoring coagulation control in these patients is highly challenging (1, 2). The favored treatments for these groups of patients are rapid reversal of coagulation factors with fresh frozen plasma (FFP), vitamin K, or coagulation factor concentrates including prothrombin complex concentrate (PCC) to prevent massive bleeding (3, 4). PCC, which contains three or four coagulation factors including factors (F) II, FVII, FIX and FX, is now widely available. It rapidly reverses these coagulation factors and corrects international normalized ratio (INR), and has a number of advantages compared to FFP, including lower volume of infusion, better safety, lower risk of transmission of blood-borne infections, no need to match the blood group, better efficacy and easier storage (5-7). Although the use of PCC for rapid reversal of oral anticoagulants is well studied (6, 8-10), there are a few studies on the efficacy of PCC to manage bleeding in patients with liver disease or patients with high INR who are scheduled to undergo surgery (11-13).

## 2. Objectives

In this study, we examined an Iranian sample and investigated the efficacy of PCC to manage bleeding in patients with liver disease and also patients with high uncorrected INR who were scheduled for surgery or biopsy.

## 3. Materials and Methods

### 3.1. Patients

This pre-post interventional study was conducted at the Shiraz University of Medical Sciences in southern Iran from February to September 2012. The aim was to evaluate the efficacy of PCC for the management of bleeding in patients without hemophilia, i.e. patients with liver disease and patients undergoing surgery or biopsy, who had high uncorrected INR and had been taking vitamin K or FFP. Based on our aim, we selected 25 patients (14 males and 11 females, mean age 18 ± 9.6 years old) by purposive, non-probability sampling method. The patients were divided into two groups. Sixteen patients had post-liver disease bleeding (group 1) and 9 patients had high uncorrected INR and were scheduled for surgery or biopsy (group 2). The PCC used in this study was Kedrion (Italy) three-factor concentrate containing human plasma coagulation FII 500 IU/vial, human plasma coagulation FIX 500 IU/vial and human coagulation FX 400 IU/vial. The patients were treated with a single dose of 25 IU/kg PCC and efficacy was defined as any reduction in, or cessation of bleeding episodes and correction of INR after treatment. Any adverse effects such as thrombosis and viral markers were recorded before and after the treatment. Prothrombin time, INR and viral markers including HBV, HCV, HIV and parvovirus B19 were tested with routine diagnostic methods. This study was approved by the Medical Ethics Committee of Shiraz University of Medical Sciences, and written consent was provided by all participants.

### 3.2. Statistical Analysis

The INR values before and after the treatment for the entire group of patients and for each treatment group were compared with paired sample t-test and Wilcoxon’s signed rank test. Normality of the data was checked by the Shapiro-Wilk test, which showed a normal distribution. P values < 0.05 were considered statistically significant.

## 4. Results

### 4.1. Efficacy

The demographic data for all patients are shown in Table 1. In all patients (n = 25) post-treatment INR decreased significantly from a mean of 4.1 ± 0.3 to 1.4 ± 0.2 (P = 0.0001). The clinical efficacy in group 1 (16 patients with liver disease including acute liver failure and atresia, cryptogenic cirrhosis, tyrosinemia, progressive familial intrahepatic cholestasis, Wilson disease and autoimmune hepatitis) was significant, and there were no bleeding episodes after the treatment. INR in this group decreased significantly after PCC treatment from 4 ± 0.3 to 1.4 ± 0.2 (P = 0.0001). 

**Table 1.** Demographic Data of Patients in the Two Groups

	Male/Female, Mean (SD)	INR before treatment. Mean (SD)	INR after treatment, Mean (SD)
**Post-liver disease bleeding**	9/7 (17 ± 5.4)	4 ± 0.3	1.4 ± 0.2
**High INR candidate for surgery or biopsy **	5/4 (18 ± 5.3)	4.3 ± 0.4	1.3 ± 0.1

In the nine patients with high uncorrected INR (group 2) who were taking FFP and vitamin K, clinical efficacy was significant without any bleeding during or after surgery. Mean INR decreased significantly from 4.3 ± 0.4 to 1.3 ± 0.1 (P = 0.008) after the treatment. Considering the estimated difference of 2, between INR before and after treatment and observed difference of 2.7 in our population, and α = 0.05, power of the study was calculated as 0.9.

### 4.2. Safety

All patients tolerated the treatment well without any significant adverse effects such as thrombosis or viral infections.

## 5. Discussion

PCC is a blood coagulation product mainly available in two types: a three-factor product containing coagulation FII, FIX and FX, and a more widely available, rebalanced four-factor product that also contains FVII (1, 14). These two types may also differ in other components such as heparin, antithrombin and proteins C and S, which are added by some manufacturers. Since vitamin K-dependent coagulation factors (e.g. FII, FVII, FIX and FX) are an important component of PCC, the benefits of PCC for vitamin K antagonists such as warfarin to reverse bleeding are well known. But a few studies are available regarding the efficacy of PCC in the management of bleeding due to causes other than hemophilia, such as liver disease and high INR in patients scheduled for surgery. In a study by Schick et al. the INR level was significantly reduced (P < 0.01) after the infusion of PCC in 50 patients, 12 of whom required urgent anticoagulant reversal and 38 of whom had severe preoperative coagulopathic bleeding. These authors also found that hemoglobin increased and mean arterial pressure stabilized after PCC therapy (3). Schochl et al. on reported a 17-year-old caucasian woman with severe trauma who was treated initially with fibrinogen and later with PCC. Treatment successfully restored hemostasis and minimized the requirement for blood transfusion products (15). Wong reported a 71-year-old man on warfarin with trauma, who was treated with PCC and vitamin K and then underwent neurosurgery without complications and had a normalized INR (16). Lorenz et al. reported that the infusion of PCC was tolerated well in 22 adult patients with bleeding before surgery or an invasive intervention for severe liver disease. Clinical efficacy was judged “very good” in 76% of patients after the first PCC treatment, without any related adverse effects (12).

Similar to other studies that evaluated the efficacy of PCC in patients without hemophilia, we found a good response to PCC treatment in patients with liver disease and also patients with high INR and at risk of bleeding who were scheduled for surgery. After treatment, INR decreased significantly in both groups (from 4 ± 0.3 to 1.4 ± 0.2 in patients with liver disease and from 4.3 ± 0.4 to 1.3 ± 0.1 in patients undergoing surgery), and none of the patients experienced any significant bleeding after the treatment. All patients tolerated the treatment well without any adverse effects. According to our findings, PCC treatment for patients without hemophilia but who have liver disease or high INR and are scheduled to undergo surgery appears to be safe and effective, and therefore may be a good treatment option for these groups of patients. Our study was limited due by its sample size however the purpose of the study was acceptable.

Our results together with earlier studies show that PCC for the management of patients without hemophilia but at risk of bleeding is clinically effective and is not associated with any major adverse effects. However, additional studies with larger groups are needed to more thoroughly evaluate the efficacy and safety of PCC, especially with regards to appropriate dosages in different groups of patients.
